# Mobile Technology in E-Learning for Undergraduate Medical Education on Emergent Otorhinolaryngology–Head and Neck Surgery Disorders: Pilot Randomized Controlled Trial

**DOI:** 10.2196/mededu.9237

**Published:** 2018-03-08

**Authors:** Li-Ang Lee, Shu-Ling Wang, Yi-Ping Chao, Ming-Shao Tsai, Li-Jen Hsin, Chung-Jan Kang, Chia-Hsiang Fu, Wei-Chieh Chao, Chung-Guei Huang, Hsueh-Yu Li, Cheng-Keng Chuang

**Affiliations:** ^1^ Faculty of Medicine Chang Gung University Taoyuan Taiwan; ^2^ Graduate Institute of Clinical Medicine Sciences Chang Gung University Taoyuan Taiwan; ^3^ Department of Otorhinolaryngology–Head and Neck Surgery Linkou Chang Gung Memorial Hospital Taoyuan Taiwan; ^4^ Graduate Institute of Digital Learning and Education National Taiwan University of Science and Technology Taipei Taiwan; ^5^ Graduate Institute of Medical Mechatronics Department of Computer Science and Information Engineering Chang Gung University Taoyuan Taiwan; ^6^ Department of Neurology Linkou Chang Gung Memorial Hospital Taoyuan Taiwan; ^7^ Department of Otorhinolaryngology–Head and Neck Surgery Chiayi Chang Gung Memorial Hospital Chiayi Taiwan; ^8^ Department of Otorhinolaryngology–Head and Neck Surgery Keelung Chang Gung Memorial Hospital Keelung Taiwan; ^9^ Department of Laboratory Medicine Linkou Chang Gung Memorial Hospital Taoyuan Taiwan; ^10^ Graduate Institute of Biomedical Sciences Department of Medical Biotechnology and Laboratory Science Chang-Gung University Taoyuan Taiwan; ^11^ Department of Surgery Linkou Chang Gung Memorial Hospital Taoyuan Taiwan

**Keywords:** e-learning, gamification, mobile technology, randomized controlled trial, video lecture

## Abstract

**Background:**

The use of mobile technology in e-learning (M-TEL) can add new levels of experience and significantly increase the attractiveness of e-learning in medical education. Whether an innovative interactive e-learning multimedia (IM) module or a conventional PowerPoint show (PPS) module using M-TEL to teach emergent otorhinolaryngology–head and neck surgery (ORL-HNS) disorders is feasible and efficient in undergraduate medical students is unknown.

**Objective:**

The aim of this study was to compare the impact of a novel IM module with a conventional PPS module using M-TEL for emergent ORL-HNS disorders with regard to learning outcomes, satisfaction, and learning experience.

**Methods:**

This pilot study was conducted at an academic teaching hospital and included 24 undergraduate medical students who were novices in ORL-HNS. The cognitive style was determined using the Group Embedded Figures Test. The participants were randomly allocated (1:1) to one of the two groups matched by age, sex, and cognitive style: the IM group and the PPS group. During the 100-min learning period, the participants were unblinded to use the IM or PPS courseware on a 7-inch tablet. Pretests and posttests using multiple-choice questions to evaluate knowledge and multimedia situational tests to evaluate competence were administered. Participants evaluated their satisfaction and learning experience by the AttrakDiff2 questionnaire, and provided feedback about the modules.

**Results:**

Overall, the participants had significant gains in knowledge (median of percentage change 71, 95% CI 1-100, *P*<.001) and competence (median of percentage change 25, 95% CI 0-33, *P*=.007) after 100 min of learning. Although there was no significant difference in knowledge gain between the two groups (median of difference of percentage change 24, 95% CI −75 to 36; *P*=.55), competence gain was significantly lower in the IM group compared with the PPS group (median of difference of percentage change −41, 95% CI −67 to −20; *P*=.008). However, the IM group had significantly higher scores of satisfaction (difference 2, 95% CI 2-4; *P*=.01), pragmatic quality (difference 1.7, 95% CI 0.1-2.7; *P*=.03), and hedonic stimulation (difference 1.9, 95% CI 0.3-3.1; *P*=.01) compared with the PPS group. Qualitative feedback indicated that the various games in the IM module attracted the participants’ attention but that the nonlinearly arranged materials affected their learning.

**Conclusions:**

Using M-TEL for undergraduate medical education on emergent ORL-HNS disorders, an IM module seems to be useful for gaining knowledge, but competency may need to occur elsewhere. While the small sample size reduces the statistical power of our results, its design seems to be appropriate to determine the effects of M-TEL using a larger group.

**Trial Registration:**

ClinicalTrials.gov NCT02971735; https://clinicaltrials.gov/ct2/show/NCT02971735 (Archived by WebCite at http://www.webcitation.org/6waoOpCEV)

## Introduction

Generalism is one of the most important aspects of the novel 6-year program of undergraduate medical education (UME) that was implemented in Taiwan in 2013. The goal of UME is to provide graduates with core knowledge and skills at the highest level of competency and then to become general physicians [1]. Clinical problems associated with otorhinolaryngology–head and neck surgery (ORL-HNS) comprise 20% to 50% of presenting complaints to a primary care provider. Therefore, educating medical students about ORL-HNS is an extremely important part of their UME. However, there have been longstanding concerns regarding the low priority assigned to ORL-HNS in the UME curriculum, and a substantial mismatch between this educational need and existing curricula has been reported to result in significant downstream effects on managing ORL-HNS problems in family medical practice [2].

Since increasing the number of hours dedicated to ORL-HNS in the classroom and hospital is not practical, novel UME requires enabling self-directed learning and augmenting learning outside the classroom [3]. The use of different learning strategies is one of the most important prerequisites of academic success [4]. Mobile technology represents the next natural frontier in the evolution of e-learning [5,6], and in this context, it has been termed *mobile technology in e-learning (M-TEL)*. Using M-TEL can result in greater educational opportunities for undergraduate medical students while simultaneously enhancing the effectiveness and efficiency of learning. However, the adoption of e-learning and M-TEL requires the alignment of new educational and economic tools [7]. A blended e-learning approach has been reported to provide a cost saving of 24% compared with traditional didactic methods [8], and therefore M-TEL may be able to bridge the gap between current educational needs and that currently provided for undergraduate medical students. The successful application of e-learning requires that it meets the needs of both the learners and program, and it should be aligned with the contexts in which it is used [8]. Furthermore, individual differences may also play an important role in the effectiveness of M-TEL. For example, learners with a field-independent (FI) cognitive style have been reported to prefer e-learning technologies and to have a better performance with hypermedia systems than field-dependent (FD) learners, because they use active approaches and make better transfer of concepts in new situations [9].

In this study, we have reported the results of a pilot study of the feasibility and qualitative evaluation of a novel interactive multimedia (IM) module versus a conventional PowerPoint show (PPS) module of e-learning using the same mobile device to teach emergent ORL-HNS disorders.

## Methods

### Study Design and Setting

**Figure 1:**
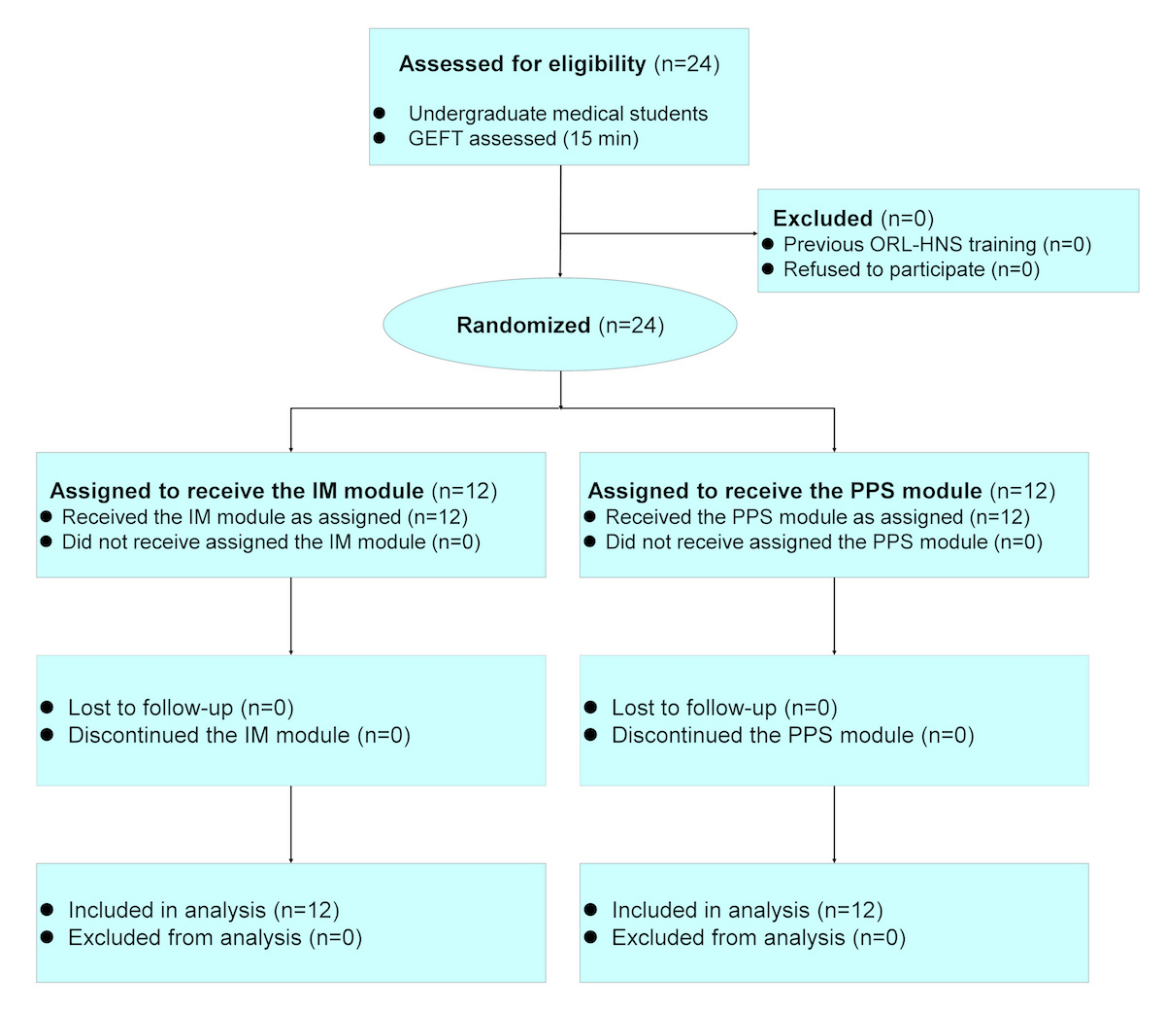
The Consolidated Standards of Reporting Trials flow diagram of this pilot study. GEFT: Group Embedded Figures Test; IM: interactive multimedia; ORL-HNS: otorhinolaryngology–head and neck surgery; PPS: PowerPoint show.

A convenience sample of 24 consecutive student volunteers were prospectively recruited according to accessibility and individuals willing to participate in the pilot study at an academic teaching hospital (Department of ORL-HNS, Linkou Chang Gung Memorial Hospital, Taoyuan, Taiwan) from November 23, 2016 to January 14, 2017. All of them had at least a basic level of computer literacy, and they were also introduced to the practical aspects of using tablets and applications. Blinding to the purpose of the prestudy during recruitment was maintained to minimize preparation bias. This study was approved by the institutional review board of Chang Gung Medical Foundation (No.: 105-5290C). Written informed consent was obtained from all participants. The study proposal was registered at ClinicalTrials.gov (Identifier: NCT02971735). The study flowchart following the Consolidated Standards of Reporting Trials (CONSORT) 2010 guidelines (Multimedia Appendix 1) [10] is shown in Figure 1.

### Establishing the M-TEL System for Emergent ORL-HNS Disorders

Emergent ORL-HNS disorders are sensitive and acute and require many consultations for the patients to receive appropriate point-of-care service and follow-up [11]. We selected the 10 most common emergent ORL-HNS disorders, including foreign body, epistaxis, ear trauma, acute otitis externa, deep neck infection, head and neck cancer and associated complications, acute otitis media, nasal trauma, acute pharyngotonsillitis, and sudden deafness (in descending order based on consultation frequency) among 300 consecutive patients who visited an otolaryngologist in 2004 at our Department of Emergency.

To design effective instructional material, we analyzed our tasks and topics and needs of 10 undergraduate students after traditional ORL-HNS lectures [12]. We then developed the instructional content using a two-round modified Delphi method. The first round included 10 academic physicians including 2 emergency physicians and 2 ORL-HNS department chiefs who designed the learning objectives and developed the instructional content according to the needs assessment. In the second round, 10 junior residents rated the relative importance of each item. We then developed a storyboard and courseware using an instructional system design model including five phases (analysis, design, development, implementation, and evaluation) [13].

**Figure 2:**
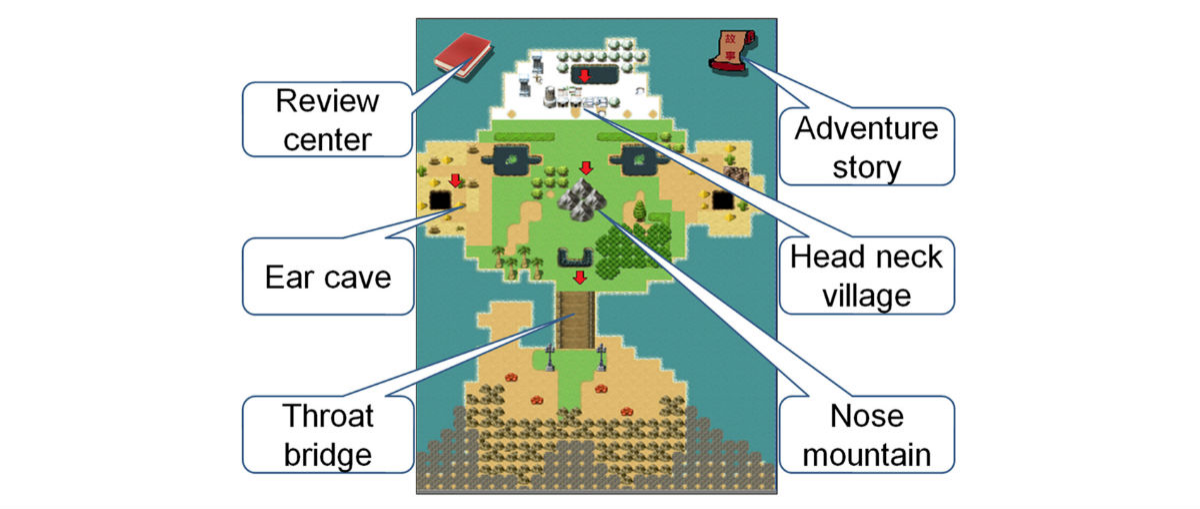
User interface of the start screen contained four instructional domains, an adventure story and a review center.

**Figure 3:**
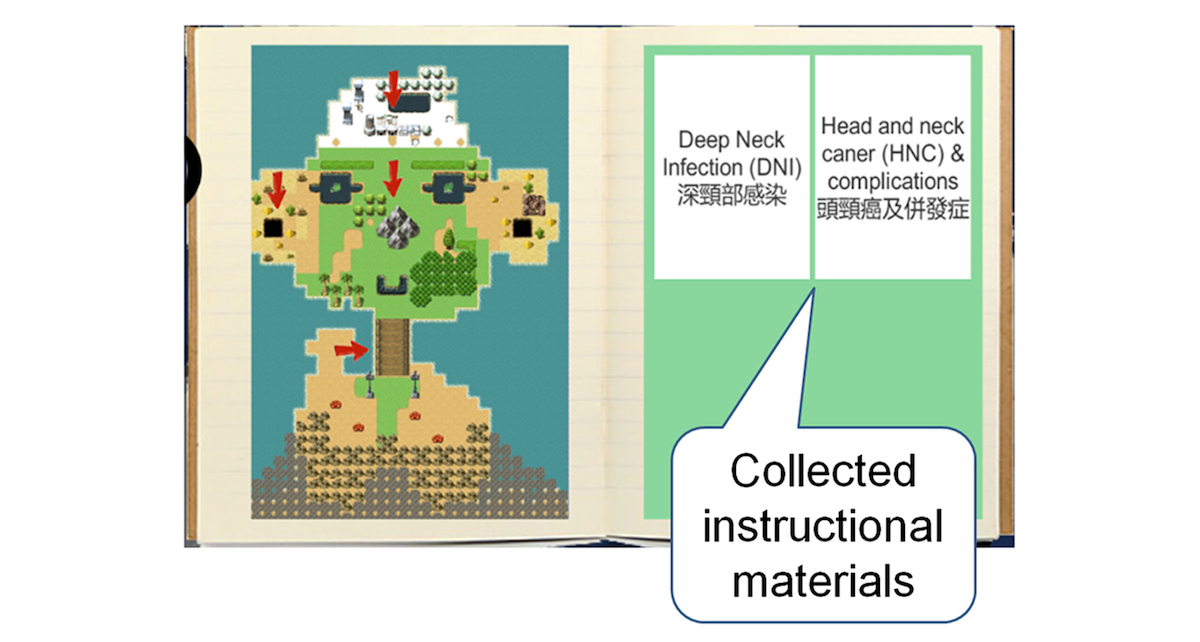
A screenshot of the review center allowing the learners to review the acquired instruction materials anytime.

Subsequently, the content was translated into an e-learning app including a novel gamified IM module and a conventional visual-auditory text-image PPS module. We created an 80-min storyboard for each module, and both modules had the same design of user interface (Figure 2). We also created a learning map to allow the learners to assess their progress in each session or their overall progress. Moreover, both modules contained simple slides for review purposes after completing the brief sessions (Figure 3).

**The PowerPoint Show (PPS) Module**

**Figure 4:**
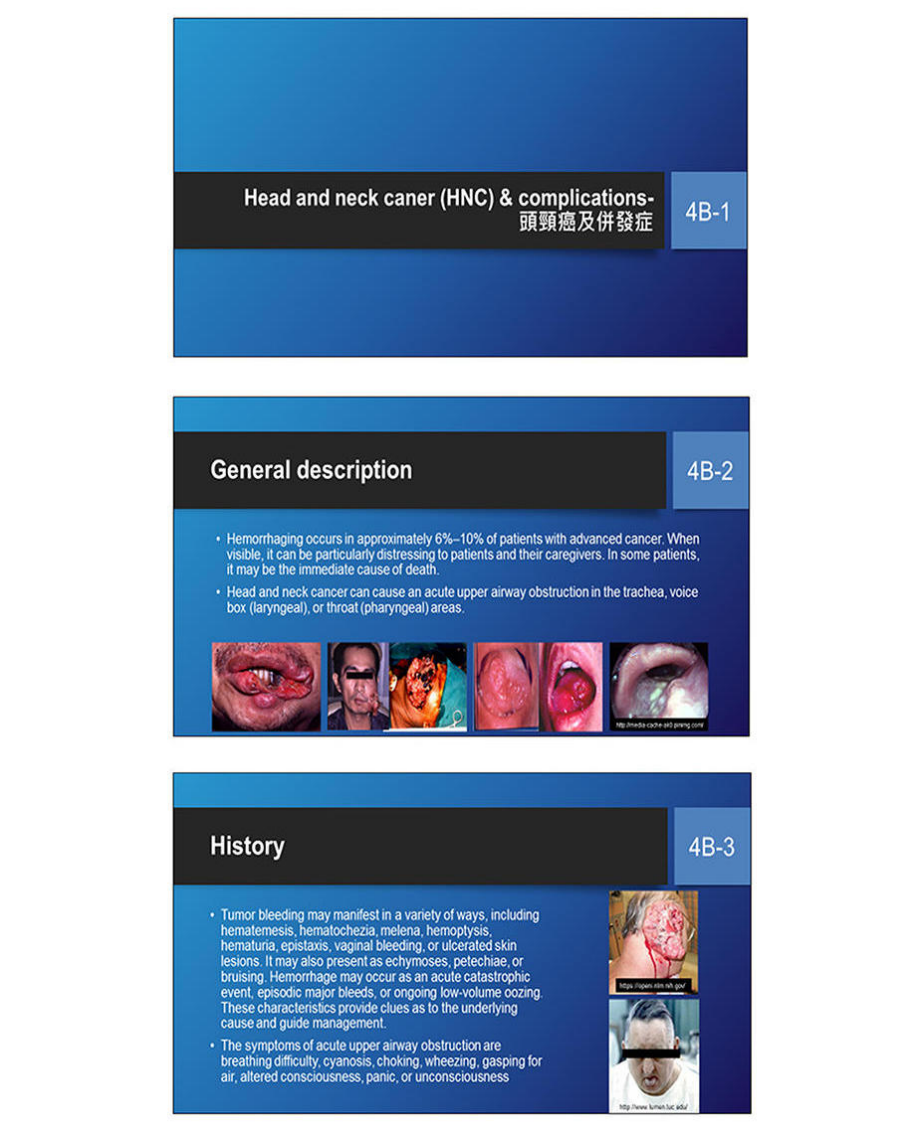
Screenshots of the PowerPoint Show module. Learners in this group needed to watch visual-auditory text-image videos including linearly arranged instructional slides (top, middle, bottom).

In the PPS module, we used video lectures to present the visual-auditory text-image context (multimedia learning) that was intended to reduce the cognitive load [14]. We recorded the PPS presentations with audio narrations and ink gestures using Camtasia Studio software version 8 (TechSmith, Okemos, MI, USA). Each mini video (6-8 min) contained seven voice and text-image slides for each disorder. We created a playback application to allow the learners to seek the videos (Figure 4).

**The Interactive Multimedia (IM) Module**

**Figure 5:**
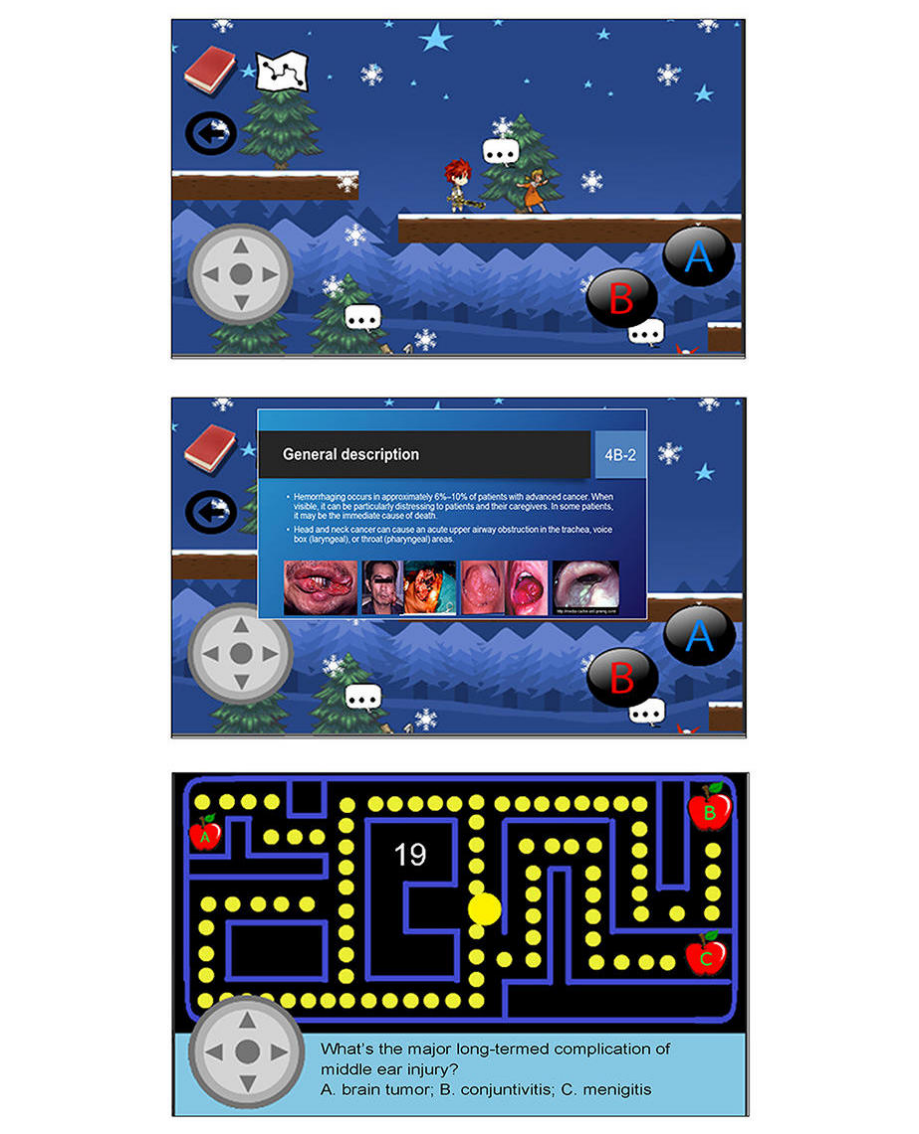
Screenshots of the interactive multimedia module. Learners in this group operated a character to run, jump, and interact with other characters (top) to obtain instructional materials (middle) and complete small game-based quizzes (bottom).

The content for the novel IM module was derived from and corresponded to the textbook-based learning material of the conventional PPS module. In the IM module, we used a game-based learning method to implement the instruction, in which the learners operated a character to run, jump, and interact with other characters (in a parkour style) to obtain learning materials (7 text-image slides per disorder) in the four domains of the 10 disorders (Figure 5). The instructional materials were briefly explained using scrolling text. After they had read the material for 2 disorders or completed 10 disorders, they had to complete small game-based quizzes that were designed to emphasize the key points and enhance their working memory [15]. Notably, contexts of the game-based quizzes were different from those of the multiple-choice questionnaires (MCQs) and multimedia situational tests (MSTs).

Two investigators from the study team reviewed the instructional content of each module using the Software Evaluation Checklist [16]. This checklist includes 7 items (curriculum connections, age/grade appreciates, investment justification, layout, support materials, instructional content, graphics/multimedia) with two (yes, no) scales (a total of 28 questions). The overall items were confirmed to be significantly correlated after computing the correlation between the 2 investigators’ reviews (Spearman correlation test, *r*=.91, *P*<.001). Major bug fixes were performed before the pilot study.

### Selection of Participants

The inclusion criteria were as follows: (1) age >20 years and (2) undergraduate medical students (defined as 3 or 4 years of medical school training [clerkship]). The exclusion criteria were (1) previous ORL-HNS training and (2) declining to participate.

**Table 1:** A general design of the multimedia situational tests.

Scenarios	Questions	Specifications of assessment
S1: Elicit history of acute otorhinolaryngology–head and neck surgery illness with an example picture.	Q1: Which is the most impossible diagnosis from four disorders?	Ability of remembering
S2: Additional symptoms and signs.	Q2: Which the less likely diagnosis from three disorders?	Ability of applying
S3: Seek critical physical findings.	Q3: Which is the most preferable diagnostic tool for further physical examination?	Ability of analyzing
S4: Interpret key physical findings of a video.	Q4: Which is the more possible diagnosis from two disorders?	Ability of analyzing
S5: Prescribe treatments according to the key features.	Q5: Which is the most effective solution?	Ability of evaluating

### Methods of Measurement

There were six different face-to-face assessments in this study.

#### Group Embedded Figures Test

The 25-item Group Embedded Figures Test (GEFT) was given to the students after enrollment to assess their cognitive style [17]. The reliability of GEFT has been confirmed (Spearman-Brown reliability coefficient, .82) [18]. The cognitive style of the learners could be determined according to the number of correct answers given by the participants. We defined a GEFT score ≤12 as *FD* and >12 as *FI* in this study.

#### Multiple-Choice Questionnaires (MCQ)

In this study, the participants were required to complete the same MCQ pretest and another different posttest immediately after the M-TEL. A 15-min 10-question standard MCQs were used to evaluate the students’ knowledge (range, 0-100) with regard to *emergent ORL-HNS disorders*. Each textbook-based MCQ was designed to be answered within 90 seconds. We established a pool of 100 MCQs and performed empirical analysis according to previous test results to determine the test difficulty and item discrimination. Before this study, the instructional content was reviewed by 2 members of staff to determine whether it was sufficient to answer all of the questions. They also used a table of specifications to ensure that there was a match between teaching and testing. Moreover, they performed judgmental analysis of the items and subsequently revised the poorly constructed items or removed the questions with an inappropriate (too easy or extremely difficult) level of difficulty. Accordingly, we constructed two different 10-question MCQ tests with the same levels of difficulty (moderate difficulty) and discrimination (good discrimination).

#### Multimedia Situational Test (MST)

The participants were required to complete the same MST pretest and another different posttest immediately after the M-TEL. The MST was a variation of the key feature test for assessments of clinical reasoning ability involving knowledge and intellectual skills (range, 0-100) [19]. A key feature was defined as a significant step in the resolution of a problem. Key feature tests were different from knowledge-based tests and can successfully predict future physician performance [20]. In this study, the MST included a set of five scenarios (a written description of a scenario with or without an image/video) for one emergent ORL-HNS disorder and five corresponding MCQs (Table 1). The MST was designed to be completed in 15 min. The two MSTs were approved by a senior member of staff to ensure the validity of the content. Evaluation of the MSTs by other students showed that internal consistency reliability was acceptable (Cronbach alpha=.76). Two members of staff confirmed that these questions could be sufficiently answered after reviewing the instructional content of the M-TEL.

#### Global Satisfaction Score

We used the global satisfaction score (GSS; range, 0-100) to measure *learner satisfaction* after the M-TEL. GSS was measured using a visual analogue scale from 0 (very dissatisfied) to 10 (very satisfied).

#### AttrakDiff2 Questionnaire

We used the AttrakDiff2 questionnaire to compare *user learning experience*. The AttrakDiff2 questionnaire was developed to evaluate the acceptance of technical innovations focusing on *user experience* [21]. The central idea behind the AttrakDiff2 is that interactive products fulfill both the pragmatic and hedonic needs of their users. It uses four qualities (attractive, identifiable, stimulating, pragmatic) with seven anchor scales (semantic differential design with a 7-point Likert-like scale) for a total of 28 questions. The mean value of an item group creates a scale value for pragmatic quality (PQ), hedonic stimulation (HQ-S), hedonic identification (HQ-I), and attractiveness (ATT). This questionnaire has been optimized to differentiate these subqualities.

#### Anonymous Feedback

We used anonymous feedback to assess *quality of learning*. Each participant in this pilot study also provided anonymous feedback about the quality of the module used after the M-TEL.

### Randomization

Figure 1 demonstrates the study flowchart. A balanced design with regard to age, sex, and cognitive style was assured by the randomization procedure. Using the Random Number Generator in IBM SPSS software (version 23; IBM, Armonk, NY, USA), computer-generated lists of random numbers were created for the allocation of the students, who were stratified by center with a 1:1 allocation using a fixed block size of 6 in both parallel subgroups. We concealed the allocation sequence from those assigning participants to intervention groups until the moment of assignment and adhered to our computer-generated randomization protocol.

### Intervention

The students were unblinded after randomization. The students in the PPS group used an app on a 7-inch tablet to watch video lectures in 10 linear-designed sessions and review the instructional materials in an ordinary office environment for 100 min. Meanwhile, the IM group played a parkour-like game to find and read the instructional materials, completed small game-based quizzes, and reviewed the instructional materials.

### Outcome Measures

The percentage change in MCQ score (ie. “knowledge gain”) after the M-TEL was the primary outcome measure. The percentage changes in MST (ie, “competence gain”), GSS, and AttrakDiff2 questionnaire scores were the secondary outcomes.

### Sample Size

There were 6 students who helped to establish and evaluate the M-TEL system for emergent ORL-HNS disorders (percentage change in MCQ: mean=31, standard deviation [SD]=16, effect size=1.94; percentage change in MST: mean=45, SD=52, effect size=0.87). In this pilot feasibility study, we needed to confirm that the students could gain knowledge and competence significantly. We estimated the sample sizes by a priori calculation (one-sample Wilcoxon signed-rank test, two-tailed, normal parent distribution, alpha=.05, power=0.95) and found that we needed at least 7 subjects for knowledge gain and at least 21 subjects for competence gain. Due to a fixed block size of 6, we determined that the sample size of the pilot study was 24.

### Statistical Analysis

Due to the relatively small sample size in the pilot study, we analyzed all variables using a nonparametric approach. Descriptive statistics were expressed as median and 95% CI. Percentage (%) changes ([after value-before value]/[before value] × 100) in the MCQ and MST were calculated. Differences between groups were analyzed using the Wilcoxon signed-rank test or the Mann-Whitney *U* test as appropriate. Categorical variables were analyzed using Fisher exact test. Effect size and 95% CI were estimated using the Hodges-Lehmann method for Mann-Whitney *U* test and Wilcoxon signed rank test and odds ratio calculation Fisher exact test to improve the quality of the reporting of our results. Statistical analyses were performed using G*Power 3.1.9.2 software (Heinrich-Heine University, Dusseldorf, Germany), Graph Pad Prism 7.00 for Windows (Graph Pad Software Inc., San Diego, CA, USA), and IBM SPSS Statistics 23.0 (IBM Corporation, Armonk, NY, USA).

## Results

### Study Participants

Twenty-four volunteers (15 males, 63%, and 9 females, 37%; median age 23 years, range 22-25 years; 21 FI, 87% and 3 FD, 13%) were recruited in the pilot study. Table 2 summarizes the variables of interest for the overall study cohort. There were no significant differences in age, sex, cognitive style, MCQ score, or MST score between the IM and PPS groups at baseline. After randomization, all participants (100%) received the intended intervention. There was no protocol deviation in the prestudy.

### Primary and Secondary Outcomes

Overall, all participants showed a significant improvement in MCQ score (ie, “knowledge gain”; median of percentage change 71, 95% CI 14-100; *P*<.001; compared with the neutral value of 0) and a significant improvement in MST score (ie, “competence gain”; median of percentage change 25, 95% CI 0-33; *P*=.007; compared with the neutral value of 0) after 100 min of learning.

The M-TEL positively impacted the GSS (median of difference 2.5, 95% CI 1.0-4.0; *P*=.002; compared with the neutral value of 5), PQ (median of difference 1.7, 95% CI 0-2.0; *P*=.003; compared with the neutral value of 0), HQ-S (median of difference 1.1; 95% CI 0.3-1.9; *P*=.04; compared with the neutral value of 0), HQ-I (median of difference 1.7, 95% CI 1.1-2.0; *P*<.001; compared with the neutral value of 0), and ATT (median of difference 1.4, 95% CI 0.9-2.1; *P*<.001; compared with the neutral value of 0).

### Differences in Outcomes Between the Interactive Multimedia (IM) and PowerPoint Show (PPS) Modules

**Figure 6:**
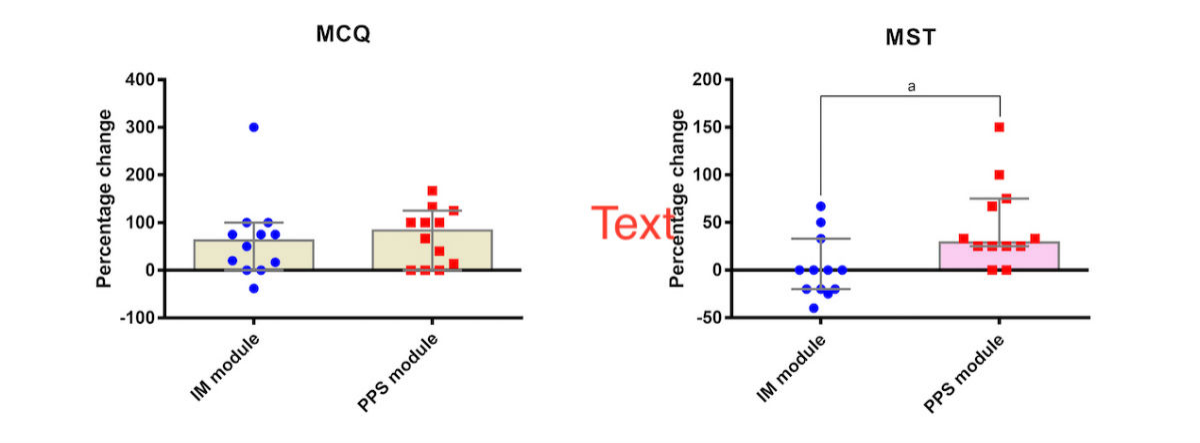
Gains of knowledge and competence. There was no significant difference in multiple choice (MCQ) test scores between the interactive multimedia (IM) and PowerPoint show (PPS) groups (left). The multimedia situational test (MST) score of the IM group was significantly lower than that of the PPS group (right). Data are expressed as median (95% CI). "a" indicates significance.

Figure 6 illustrates comparisons of the IM and PPS modules with regard to knowledge and competence gains. The PPS group had significant improvements in knowledge (median of difference of percentage change 25, 95% CI 0-40; *P*=.007) and competence (median of difference of percentage change 20, 95% CI 20-40; *P*=.005), whereas the IM group had a significant improvement in knowledge (median of difference of percentage change 25, 95% CI 0-40; *P*=.01) but not competence (median of difference of percentage change 0, 95% CI −20 to 20; *P*=.78).

**Figure 7:**
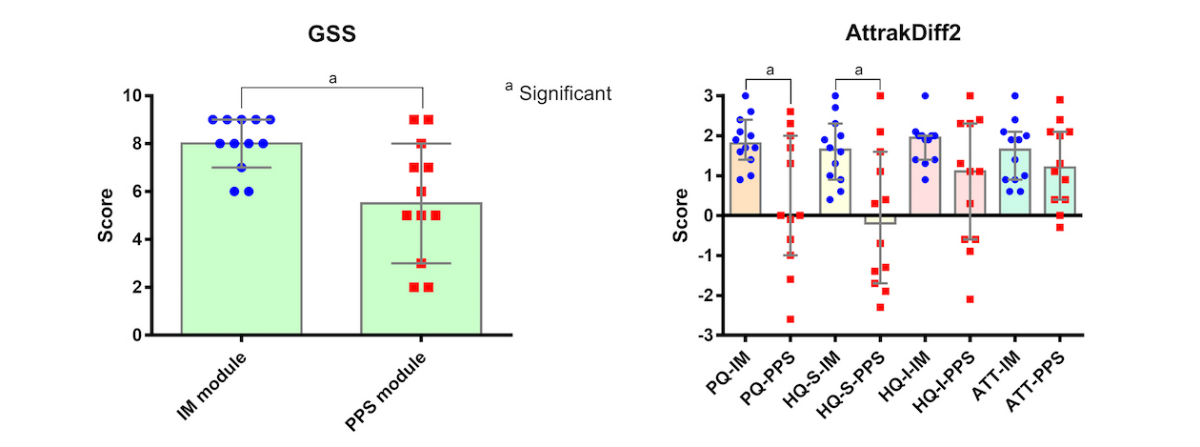
Satisfaction and learning experience. Global satisfaction score (GSS) of the IM group was significantly higher than that of the PowerPoint show (PPS) group (left). Using the AttrakDiff2 questionnaire, pragmatic quality (PQ) and hedonic stimulation (HQ-S) in the IM group were significantly higher than those of the PPS group. There were no significant differences in hedonic identification (HQ-I) and attractiveness (ATT) between the two groups (right). Data are expressed as median (95% CI). "a" indicates significance.

Although the percentage change in MCQ was not significantly different between the two groups (median of difference −24, 95% CI −75 to 36; *P*=.55), the percentage change in MST in the IM group was significantly lower than that in the PSS group (median of difference −41, 95% CI −67 to −20; *P*=.008). Figure 7 illustrates comparisons of the IM and PPS modules with regard to satisfaction and learning experience. However, the IM group had significantly higher GSS (median of difference 2, 95% CI 0-4; *P*=.01), PQ (median of difference 1.7, 95% CI 0.1-2.7; *P*=.03), and HQ-S scores (median of difference 1.9, 95% CI 0.3-3.1; *P*=.01) compared with the PPS group.

### Qualitative Feedback

The qualitative feedback from the PPS group emphasized that they found the PPS module “easy to use and follow,” “clear layout,” “enhanced knowledge,” “suitable small sessions,” and “simulated lectures.” However, they also reported that the module was “tedious,” “hypnogenetic,” and “difficult to play back.” The IM group reported that the IM module was “fun learning (attractive),” contained “enjoyable small game-based quizzes,” and was an “amazing learning experience.” However, they also considered it “difficult to use and follow,” that it contained “nonlinear instructional materials” and “some tough games.”

**Table 2:** Demographic data, cognitive style, learning outcomes, and satisfaction.

Variables		Overall, N=24	Interactive multimedia group, N=12	PowerPoint show group, N=12	Effect size, median of difference (95% CI) or odds ratio (95% CI)^a^	*P* value^b^
**Demographics**						
	Age in years, median (95% CI)	23 (22-23)	23 (22-23)	23 (22-24)	0 (−1 to 0)	.32
	Male sex, n (%)	15 (63)	7 (58)	8 (67)	−0.09 (−0.49 to 0.32)	>.99
**Cognitive style**						
	Group Embedded Figures Test score, median (95% CI)	18 (17-18)	18 (15-18)	17 (17-18)	0 (−1 to 1)	.80
	Field-dependence, n (%)	3 (13)	2 (17)	1 (8)	−0.13 (−0.54 to 10.28)	>.99
**Learning outcomes**						
	Multiple-choice question_before, median (95% CI)	40 (40-50)^c^	40 (40-50)^c^	40 (30-60)^c^	5 (−10 to 10)	.52
	Multiple-choice question_after, median (95% CI)	70 (60-80)^c^	70 (50-80)^c^	70 (60-80)^c^	0 (−10 to 10)	.71
	Percentage change in multiple-choice question, median (95% CI)	71 (14-100)^d^	63 (0-100)	84 (0-125)	−24 (−75 to 36)	.55
	Multimedia situational test_before, median (95% CI)	80 (60-80)^c^	80 (60-100)	70 (40-80)^c^	20 (0-20)	.13
	Multimedia situational test_after, median (95% CI)	80 (80-100)^c^	80 (60-80)	90 (80-100)^c^	−20 (−20 to 0)	.02
	Percentage change in multimedia situational test, median (95% CI)	25 (0-33)^d^	0 (−20 to 33)	29 (25-75)	−41 (−67 to −20)	.008
**Learning satisfaction**						
	Global satisfaction score, median (95% CI)	8 (6-9)^d^	8 (7-9)^d^	6 (3-8)	2 (0-4)	.01
**Learning experience (AttrakDiff2 questionnaire)**						
	Pragmatic quality, median (95% CI)	1.7 (0-2.0)^d^	1.8 (1.4-2.4)^d^	0 (−1.0 to 2.0)	1.7 (0.1-2.7)	.03
	Hedonic stimulation, median (95% CI)	1.1 (0.3-1.9)^d^	1.7 (0.9-2.3)^d^	−0.2 (−1.7 to 1.6)	1.9 (0.3-3.1)	.01
	Hedonic identification, median (95% CI)	1.7 (1.1-2.0)^d^	2.0 (1.4-2.0)^d^	1.1 (−0.6 to 2.3)	0.8 (−0.3 to 2.3)	.18
	Attractiveness, median (95% CI)	1.4 (0.9-2.1)^d^	1.7 (0.9-2.1)^d^	1.2 (0.4-2.1)^d^	0.2 (−0.5 to 1.0)	.59

^a^Effect sizes were calculated with the use of Hodges-Lehmann method for Mann-Whiney *U* test and Wilcoxon signed-rank test, or odds ratio calculation for Fisher exact test.

^b^Mann-Whiney *U* test (continuous variables) or Fisher exact test (categorical variables).

^c^*P*<.05, before versus after, Wilcoxon signed-rank test (two-tailed).

^d^*P*<.05, compared with a neutral value (“0” for multiple-choice question and multimedia situational test, or “5” for “global satisfaction score” or “0” for “ArakDiff2”), Wilcoxon signed-rank test (two-tailed).

## Discussion

### Principal Findings

To the best of our knowledge, this is the first study to investigate the benefits of M-TEL to improve knowledge and competence of emergent ORL-HNS disorders. Our findings indicate that using well-designed M-TEL instructional materials can help undergraduate medical students to reinforce their existing knowledge (intermediate effect) and competence (small effect) of such a sensitive and important subject and provide an enjoyable learning experience (small-to-intermediate effect). In addition, our findings suggest that an IM module has the potential to provide an instructional approach to enhance knowledge as effectively as a PPS module. Although the PPS module was superior to the IM module with regard to competence gained (small effect), the students preferred the IM module to the PPS module because of it being more efficient and enjoyable to use (small-to-intermediate effect). However, qualitative feedback recommended that both modules needed to have better quality of design and function. Since the development of the IM module was more time-consuming (3 months vs 1 months) and more expensive (US $12,500 vs US $2500) than that of the PPS module, the IM module needs to be further improved with regard to competence gain in the future. For example, we can modify the IM model according to the teaching strategies and principles of instructional design and pedagogy used in virtual patient cases to support the development of clinical reasoning skills [22].

### Limitations

Some caveats of our study merit comment. First, we included a convenience sample which may have led to exclusion bias. A more even distribution of the cognitive styles will provide more accurate data. However, FD volunteers are not frequently encountered in our undergraduate medical students (less than 10%). Moreover, it is very difficult to perform probability sampling at a regular medical school. Second, we did not investigate social interaction, self-motivation, and self-regulation (important elements of e-learning) in detail [23-25]. The effects of M-TEL on these factors during the learning process will be closely monitored when the M-TEL app is made available to the students.

### Comparison With Prior Work

The role of M-TEL, especially as it pertains to the undergraduate medical student, is evolving. It is superior to classical learning in that it provides opportunities to learn outside of the classroom via the Internet and computer software [26]. A systematic review of the impact of e-learning for undergraduate medical students suggested that e-learning is equivalent and possibly superior to traditional learning regarding knowledge, skills, attitude, and satisfaction [27,28]. Our preliminary results are similar to previous studies, in that the interactive elements of M-TEL for medical education could facilitate learning complex topics with promising results in terms of knowledge gain and attitude [29-33]. However, M-TEL may not be an approach that is suitable for all [34].

Most previous studies have compared interactive e-learning with text-based learning or classroom lectures. Fundamental differences among these learning methods such as a learner-focused design [35] and unlimited learning place/time [36] may be confounding factors. The focus of an M-TEL course is the learner, since there is no instructor. Therefore, the developers need to understand the knowledge base (needs assessment) and learning preference of the learners when establishing the module [4,35]. Learning preferences are conscious and intentional strategies to achieve well-defined ends and include three layers: core (cognitive style), intermediate (information processing), and external (instructional preference) [37]. Since learning preferences are often observed to favor rewarded responses to high frequency or high likelihood questions [38], an M-TEL course needs to clearly explain the required information to the learner. E-learning has moved into a more student-centered model in a systematic review [9]. Therefore, it is better to take the learner’s individual cognitive style and instructional preference into consideration in the development of the M-TEL. Moreover, significant differences in perceived ease of use, external control, behavioral intention, and use of e-learning between males and females have been reported when adopting an e-learning platform [39]. Although not statistically significantly different in this pilot study, cognitive style and gender of the participants should be controlled in randomized controlled trials.

Unlike traditional classroom lectures, learners can start and stop M-TEL (or text-based learning) at any time or place of their choosing [36]. When M-TEL learners want to review instructional content, they can immediately do so and reduce the errors involved in teaching and learning. Of note, this may be unfair to students only receiving classroom instruction, because M-TEL learners can study when they are most receptive and spend more time to comprehend the learning materials. In this study, the PPS module was similar to the online learning and flipped classroom, and the learners could choose and review the content by themselves. Despite the relatively low reported level of satisfaction, the PPS module was more familiar to our students with a lower cognitive load, and this allowed a deeper understanding to facilitate superior competence gain compared with the IM module. Since the majority of our subjects had FI cognitive style, it could have an impact on the outcome of competence gain. In the past, Bertini et al [40] found that FI learners are more likely to be worse at “tests requiring learners to recall information in the form or structure that it was presented” than FD learners. In the study, MSTs have been designed for evaluating competence with regard to the clinical reasoning process. The nonlinear structure of presentation of the IM module might limit FI learners to recall information to answer a 5-question MST. However, interactive game-based learning seems to be a promising didactic tool to achieve higher long-term knowledge retention [41].

### Conclusions

The use of different learning strategies is one of the most important prerequisites of academic success among undergraduate medical students and can lead to a positive attitude toward learning [4]. M-TEL using an IM module seems to be an effective, enjoyable, and pragmatic way to instruct emergent ORL-HNS disorders in undergraduate medical students. However, results from this pilot study suggest that instructors may need to provide other learning methods to reinforce students’ competency. While the small sample size reduces the statistical power of our results, especially with regard to cognitive style, its design seems to be appropriate to determine the effects of M-TEL using a larger group.

## Appendix

Multimedia Appendix 1 CONSORT-EHEALTH checklist (V 1.6.1).

## Abbreviations

ATT: attractiveness
CONSORT: Consolidated Standards of Reporting Trials
FD: field-dependent
FI: field-independent
GEFT: Group Embedded Figure Test
GSS: global satisfaction score
HQ-I: hedonic identification
HQ-S: hedonic stimulation
IM: interactive multimedia
MCQ: multiple-choice questionnaire
MST: multimedia situational test
M-TEL: mobile technology in e-learning
ORL-HNS: otorhinolaryngology–head and neck surgery
PPS: PowerPoint show
PQ: pragmatic quality
UME: undergraduate medical education
